# Exploring the Quality of Dynamic Open Government Data Using Statistical and Machine Learning Methods

**DOI:** 10.3390/s22249684

**Published:** 2022-12-10

**Authors:** Areti Karamanou, Petros Brimos, Evangelos Kalampokis, Konstantinos Tarabanis

**Affiliations:** Information Systems Lab, Department of Business Administration, University of Macedonia, 54636 Thessaloniki, Greece

**Keywords:** open government data, dynamic government data, high-valuable data, real-time data, traffic data, data quality, isolation forest, eXplainable artificial intelligence

## Abstract

Dynamic data (including environmental, traffic, and sensor data) were recently recognized as an important part of Open Government Data (OGD). Although these data are of vital importance in the development of data intelligence applications, such as business applications that exploit traffic data to predict traffic demand, they are prone to data quality errors produced by, e.g., failures of sensors and network faults. This paper explores the quality of Dynamic Open Government Data. To that end, a single case is studied using traffic data from the official Greek OGD portal. The portal uses an Application Programming Interface (API), which is essential for effective dynamic data dissemination. Our research approach includes assessing data quality using statistical and machine learning methods to detect missing values and anomalies. Traffic flow-speed correlation analysis, seasonal-trend decomposition, and unsupervised isolation Forest (iForest) are used to detect anomalies. iForest anomalies are classified as sensor faults and unusual traffic conditions. The iForest algorithm is also trained on additional features, and the model is explained using explainable artificial intelligence. There are 20.16% missing traffic observations, and 50% of the sensors have 15.5% to 33.43% missing values. The average percent of anomalies per sensor is 71.1%, with only a few sensors having less than 10% anomalies. Seasonal-trend decomposition detected 12.6% anomalies in the data of these sensors, and iForest 11.6%, with very few overlaps. To the authors’ knowledge, this is the first time a study has explored the quality of dynamic OGD.

## 1. Introduction

The open government data (OGD) movement, which urged governments and public organizations to open up their data for others to reuse for both private and commercial purposes, emerged at the beginning of the 21st century [1]. Innovative political initiatives such as the Public Sector Information (PSI) Directive in Europe in 2003 [2] and the U.S. President’s Obama open data program in 2009 [3] supported this movement and made OGD a political priority. In the next years, numerous OGD portals at various administrative levels (e.g., local, regional, national, etc.) and in many countries across the globe were launched [1,4,5,6].

The expected benefits for citizens, businesses, and public administration and the potential impact on the society as a whole have been extensively described in the literature. OGD are expected to strengthen transparency [7] and improve decision-making processes [8], stimulate economic growth and innovation [9,10], and provide opportunities for the development of more effective public services [11,12], including Integrated Public Services (IPS) [13]. However, so far, the impact of OGD is still rather limited [14], with many studies exploring the reasons that hinder open government initiatives from reaching their full potential [6].

OGD is a rapidly evolving phenomenon. The amount of OGD produced and disseminated increases exponentially, and new types of data are being generated. For example, dynamic data (including environmental, traffic, satellite, meteorological, and sensor-generated data) have been recently recognized by the European Commission as an important part of OGD, presenting huge potential economic value [15]. Due to the suitability of dynamic OGD for the creation of value-added services, applications and high-quality and decent jobs, they are recognized as high-value data (HVD), whose reuse has significant societal, environmental, and economic benefits [15]. The collection and dissemination of this type of data impose new requirements and challenges. For example, dynamic data are characterized by their high variability and rapid obsolescence, making their immediate availability and regular updates crucial for the creation of added-value services and applications. Moreover, sensors are prone to malfunctions caused by, e.g., bad weather and temperature conditions, resulting in anomalous observations [16]. As a result, anomaly detection and classification methods need to be applied to detect and correct corrupt or inaccurate records. Although the quality of OGD has been recognized as being of vital importance, related works (e.g., [6,17,18]) tend to focus more on the quality of the metadata and not on the data itself.

At the same time, innovative data analysis and exploitation methods for OGD emerge, such as artificial intelligence [19], including machine learning [20]. These technologies can provide opportunities for the creation of innovative and intelligent services and applications, which are built upon the use and combination of new types of OGD. These applications are considered “data intelligence applications”. For example, data intelligence applications exploiting traffic data involve predicting various traffic states such as traffic flow, traffic speed, and traffic demand [21].

Hence, in order to be able to better understand and achieve the economic and social potential offered by the reuse of OGD, we need to keep pace with those rapid changes and investigate the exploitation of new types of OGD employing state-of-the-art technologies.

The objective of this paper is to explore the quality of Dynamic Open Government Data in order to facilitate the development of effective, efficient, and entrusted data intelligence applications. To this end, we focus on and study a single case, namely the traffic data of the Region of Attica that are provided through data.gov.gr (accessed on 24 October 2022), the official Greek OGD portal. This case was selected because it involves the use of an API for accessing HVD for traffic, which allows for immediate availability and regular updates of the data. Specifically, traffic data are updated hourly in the Greek OGD portal.

Our research approach involves the exploration and evaluation of the provided data regarding the existence of missing values and anomalies. Anomaly detection comprises both the identification of (i) anomalous flow-speed correlations and (ii) deviations from the normal traffic pattern (see Section 2.2 for details). These two present complementary views of the traffic data quality. Deviations from the normal traffic pattern are detected using a statistical method and an unsupervised machine learning method with one variable. Detected deviations are also classified in sensor faults and unusual traffic conditions. The quality of traffic data is further explored by implementing a second machine learning scenario that uses multiple variables.

This paper is organized as follows: Section 2 presents the background knowledge required to understand the content of this work. Section 3 describes in detail the steps followed in this research. Section 4 provides the details of the case vignettes of this work and explores traffic data. Then, Section 5 detects the missing values in the traffic data, while Section 6 detects anomalies in the data using three methods, namely flow-speed correlation analysis (Section 6.1), seasonal-trend decomposition using Loess (Section 6.2), and isolation forest (Section 6.3). It also classifies detected anomalies as sensor faults and unusual traffic conditions. Thereafter, Section 7 explores anomalies in the traffic data using multiple variables. Finally, Section 8 discusses the results of this work, and Section 9 concludes this study.

## 2. Background

This section presents the background knowledge required to understand the content of this paper. Specifically, it describes dynamic Open Government Data, including open traffic data, the anomaly detection methods used in this work, and anomaly explanation.

### 2.1. Dynamic Open Government Data

Open Government Data (OGD) are data published by the public sector, freely distributed to all citizens without further restrictions [22]. The term “open” refers to any data or information that can be equally and freely used, modified or shared by all citizens and businesses [11,23]. Today, a large number of public authorities and National Statistical Institutes internationally have already demonstrated their commitment to opening their data by launching OGD portals (e.g., the European Data Portal (https://data.europa.eu/ accessed on 25 October 2022)). OGD portals provide a plethora of datasets. For instance, the European Data Portal provides 1,538,031 datasets describing data that can be classified into 13 themes, including transport, economy and finance, environment, and population and society. In total, 0.75% of the datasets (10,667) come from Greece.

Dynamic data (including environmental, traffic, satellite, meteorological and sensor-generated data) have been recently recognized as an important part of OGD [15]. The importance of dynamic data, also mentioned as real-time data, has already been stressed in the literature (e.g., [24]). Unlike static data, dynamic data are characterized by their high variability and rapid obsolescence, making their immediate availability and regular updates crucial for the creation of added-value services and applications. Hence, the transmission of dynamic data should be possible immediately after their collection via an Application Programming Interface (API).

#### Open Traffic Data

Dynamic (or real-time) data with traffic-related information (e.g., counted number of vehicles, average speed) generated from sensors are increasingly provided by Open Government Data (OGD) portals. This type of data facilitates the delivery of public services in the Smart City context, improving the quality of citizens’ life and stimulating economic growth [25].

Open Traffic Data are usually aggregated by minute or hour and are available in various standard formats (e.g., using the JavaScript Object Notation—JSON, or eXtensible Markup Language, XML formats). However, that data are not always provided in real-time and cannot easily be retrieved to an external solution using an Application Programming Interface (API) [26]. Specifically, only a few of the OGD portals use an Application Programming Interface (API) to enable accessing and retrieving the data, hampering their use in data intelligence applications. In addition, only a few of the OGD portals provide streaming traffic data, i.e., updated traffic measurements every minute, while the rest of them update data every 5 min, 1 h or 1 day. Finally, some of them only provide access to historical data.

For instance, the Norwegian Public Roads Administrations’ Traffic Data API (https://www.vegvesen.no/trafikkdata/api accessed on 25 October 2022) provides hourly aggregated traffic data from the roads in Norway. The API can be accessed via a Graphical User Interface (GUI) or via programming languages to obtain data such as traffic volumes, spatial information, and number of lanes. The provided historical data trace back to 2019. The Swedish OGD portal (https://api.trafikinfo.trafikverket.se/ accessed on 25 October 2022) provides a GUI for querying streaming traffic data with the POST method for API. The data are returned in XML or JSON format and contain traffic flow data, arrivals and departures of ferries, road condition information, weather information etc. The streaming data are updated every minute. Historical data cannot be accessed through the API. In addition, the open API from Helsinki (https://helsinki-public.azurewebsites.net/ accessed on 25 October 2022) provides sensor traffic such as the volume of vehicles, their average speed and type. The open API is updated every 5 min and returns data from the past hour aggregated in 5 min, 1 h, or 1 day intervals in JSON format. Furthermore, a promising OGD initiative is provided by the government of Switzerland (https://opentransportdata.swiss/en/cookbook/rt-road-traffic-counters/ accessed on 25 October 2022), where various road authorities record traffic movements in a grouped, summarized dataset, which is freely accessible. The OGD traffic dataset contains traffic measures from the country’s most important roads near significant urban regions and national highways. The dataset uses the DATEX II, a standard for the exchange of road traffic data based on a specific XML schema. The advantage of this portal is that it returns real-time streaming data every minute. New incoming data replace the last published data, while historical data are not available in this portal. To the authors’ knowledge, the Swiss OGD portal is the only European open portal that provides streaming data using 1 min intervals. The data can be downloaded in JSON format with an http POST request using an API key (token) after a user’s registration. Finally, this study examines the quality of sensor-generated data from the Greek OGD portal (https://www.data.gov.gr/datasets/road_traffic_attica/ accessed on 25 October 2022). Provided traffic data include hourly aggregated information for traffic flow and average speed can be downloaded in CSV or JSON format but can also be accessed via an API using authorization keys (tokens). Traffic data are updated hourly in the portal.

### 2.2. Anomaly Detection

Sensors are prone to malfunctions caused by, e.g., bad weather, temperature conditions, or damages, resulting in anomalous observations. There is a plethora of definitions in the data mining and statistics literature for anomalies. For instance, they have been defined as outliers, abnormalities, discordants, or deviants [27] and as observations that do not follow the well-defined normal behavior of data [28]. Under the assumption that the majority of observations in a dataset are normal, anomalies only represent a very small proportion of the dataset, and their distribution significantly deviates from the distribution of the rest of the data [29]. In this direction, anomaly detection refers to the research area and techniques that detect data points that deviate from the dataset by calculating the likelihood of this point being an anomaly, also called an anomaly score. Finally, a threshold value is defined so that points with an anomaly score greater than this threshold are considered anomalies. Therefore anomaly detection problems are defined as binary label classification tasks determining whether an observation is normal or an anomaly. Anomaly detection has a wide range of applications in several domains, such as intrusion detection for computer-based systems, fraud detection for banks and insurance companies, and medical anomaly detection for health monitoring systems [30]. In the Internet of Things (IoT) domain, sensor monitoring process demands high quality and trustworthy detection of corrupt sensor data, ensuring the quality of the transmitted data. In-situ sensors and wireless sensor networks (WSN) produce large sequences of observations. Therefore, anomaly detection for sensor-generated data is strongly linked with time-series analysis and forecasting.

There are two main categories for sensor anomaly detection: simple statistical or time-series analysis techniques and machine learning techniques. The former refers to statistical approaches for time series anomaly detection such as regressive models ARIMA, moving average, HA [31] and time series decomposition techniques such as the seasonal-trend decomposition. The latter refers to machine learning methods that learn specific patterns from the time-series data and are further categorized into supervised and unsupervised approaches. Supervised anomaly detection methods are applied on labeled time series datasets, such that for each observation (timestamp), the label is known (anomaly or normal data point), and the dataset contains both normal points and outliers. Examples of supervised anomaly detection algorithms are neural networks LSTMs (Long Short Memory Networks) [32] and one-class SVM (Support Vector Machines) [33]. On the contrary, unsupervised anomaly detection methods assume that time-series data are unlabeled. Most of the unsupervised approaches use distance-based methods or auto-encoders (neural networks that reconstruct the input data) [34]. Finally, apart from statistical and machine learning methods, several domain-oriented thresholds are defined in order to determine whether an observation is considered anomalous. In our case, this threshold is defined as the flow-speed correlation value, the maximum number of vehicles that can pass a specific counting point (a sensor) in a particular time interval.

In our research approach, anomaly detection comprises both the identification of (a) anomalous flow-speed correlations and (b) deviations from the normal traffic pattern using a statistical time-series method and an unsupervised machine learning algorithm. These two present complementary views of the traffic data quality. To this end, we employ the following methods, respectively.

#### 2.2.1. Anomalous Flow-Speed Correlation

In traffic data, the number of vehicles counted by a sensor and their average speed are strongly correlated. In particular, considering that each sensor measures data that pass from one or more lanes, the maximum number of vehicles that can pass in all lanes in one hour can be calculated as [35]:

$$number\_of\_vehicles=\frac{average\_speed*1000}{average\_vehicle\_length+\frac{average\_speed}{3.6}}*number\_of\_lanes$$

where *average_speed* is the average speed provided by the sensors measured in km per hour and *average_vehicle_length* is the average length of the different types of vehicles, the fraction *average_speed*/3.6 represents the “safe driving distance” that should be kept between vehicles and is based on the vehicle speed, and *number_of_lanes* is the number of lanes in the road each sensor is positioned. The value of *average_vehicle_length* is set to four. When the number of vehicles measured by a sensor in an hour is higher than this value, then the measurement is considered an anomaly.

#### 2.2.2. Seasonal-Trend Decomposition Using Loess for Anomaly Detection

The seasonal-trend decomposition of periodic time series using Loess (STL) is a fundamental method for time series analysis, with many applications in anomaly detection and forecasting [36]. The robust STL algorithm performs seasonal-trend decomposition using the statistical smoother “locally estimated scatter plot smoothing”-“Loess” (a generalization of the moving average technique) and locally-weighted regression functions to decompose the time series. Specifically, STL considers the original time series as a composition of three components (additive model):

(1) $$y_{t}=T_{t}+S_{t}+R_{t}$$

where $y_{t}$ is the observed data at time *t*, $T_{t}$ denotes the trend in time series, $S_{t}$ is the seasonal component of the original time series, and $R_{t}$ denotes the remainder component. STL employs a statistical smoother called the loess (locally estimated scatter plot smoothing, a generalization of moving average technique), using locally-weighted regression functions to decompose the time series. The trend component shows a general pattern in time series on a long-term basis, the linear increasing (uptrend) or decreasing (downtrend). Furthermore, the seasonal component refers to the repeating patterns (periodic patterns) over time. Finally, the remaining variations in time series are the remainder component, also known as the noise. The remainder component is calculated by subtracting the trend and seasonal components from the original series. The remainder curve indicates the existence of noise present in the data. The decomposition procedure consists of the following steps, which are repeated iteratively until a final convergence [36]:

The first step is the noise removal from the time series using bilateral filtering, where neighbors with similar values are used to smooth the time series. The literature proposes a filter window of length 2*H* + 1 and filter weights computed by the Gaussian filter functions [37,38,39]. After denoising, the trend extraction is employed by modeling the trend difference using Least Absolute Deviations (LAD) and l1 regularizations. The trend is computed through the LAD loss function as an optimization problem by minimizing the sum of absolute deviations (such as the least square technique). After the trend removal from the time series, the seasonal extraction is performed by a non-local seasonal filtering. The seasonality component is defined as a weighted linear combination of the 2H+1 neighbors of $y_{t}$. The K-neighborhood technique for seasonality extraction indicates that data points with similar seasonality to $y_{t}$ will be given larger weights based on their distance to $y_{t}$ in the time dimension. As mentioned previously, these steps are performed iteratively by the STL algorithm until convergence. After the final iteration, the results of the STL decomposition algorithm are three time series and three independent graphs representing seasonal and residual trends.

STL decomposition is very useful for anomaly detection in time series by analyzing the residual curve of the STL output time series. For that reason, after the decomposition procedure, the remainder curve is divided into an area of normal data points and an area of outliers or anomalies. The limits of these areas in the remainder curve can be defined by various methods, such as the interquartile range (IQR) method or the empirical rule for normal distribution [40].

#### 2.2.3. Unsupervised Anomaly Detection with Isolation Forest

The recent development of machine learning and Big data analytics has introduced a wide variety of methods for anomaly detection. Moreover, unsupervised learning methods seem to be significantly useful for anomaly detection problems due to their ability to learn patterns from unlabeled data. Since the majority of real-world datasets do not contain labeled anomalous data, unsupervised learning approaches are a suitable choice. Unsupervised learning models do not learn patterns on labeled data but perceive the data structure in order to classify it into a particular set of classes. In the anomaly detection context, they assume that only a very small proportion of the dataset is anomalous and data groups of similar instances are considered normal. Ref. [41] performs anomaly detection using the unsupervised approach for detecting anomalies on surveillance videos while [42] detects anomalies in hyperspectral images with an unsupervised Deep Belief Network. Furthermore, ref. [43] uses an unlabeled Satellite dataset to detect outliers, implementing a deep auto-encoder technique, and ref. [44] demonstrates a method based on unsupervised learning for detecting noise on acoustic sensors.

The isolation Forest (iForest) is an anomaly detection algorithm based on the hypothesis that outliers are always rare and a few data points among the whole dataset (far from the center of normal clusters) [45]. This ensemble method isolates the outlier data points from the rest of the data, by portioning the data set using isolation trees (binary trees). Each node of an isolation tree consists of a randomly selected attribute A and a split value S, such that S A, while S corresponds to the minimum or maximum value of the selected attribute. The general idea is that anomalous data points are very different and easier to divide than the normal data points, and they are also closer to the root nodes of the isolation trees [46,47]. After the creation of a completed forest by generating T random isolation trees, the path length *h*(*x*) to isolate a data point *x* is calculated using the average number of the path lengths to isolate *x* from each isolation tree. The anomaly score is defined as:

(2) $$s\left(x\right)=2^{-\frac{E[(h(x)]}{c(n)}},$$

where c(n) is the average value of paths h(x) that is defined as:

(3) $$c\left(n\right)=2H\left(n-1\right)-\frac{2(n-1)}{n},$$

where *H* is the harmonic number, and it can be estimated by ln(i) + 0.5772156649 (Euler’s constant) [45]. If the anomaly score is closer to 1 the data point is considered an anomaly, *x* is an isolated point, and the path h(x) is relatively small. On the other hand, when h(x) is large, then *x* is not an isolated point and the anomaly score tends to 0; thus, the data point is considered nominal [45,48].

### 2.3. Anomaly Explanation

Machine learning models are complex algorithms, often referred as “black box”, that in many cases do not provide enough explanations regarding their predictions so that human users can understand. During the last decade, there has been high concern among the research community about the lack of transparency, accountability and interpretability of complex machine learning predictive models that might have consequences in many domains [49]. Therefore, under the recent development of eXplainable Artificial Intelligence (XAI), a second post-hoc model is created that explicitly explains the first predictive “black box” model. XAI has been recently employed not only in Open Government Data (OGD) [20] but also in healthcare [50,51] and transport data [52]. This new field of XAI has applications in the anomaly detection research area named as eXplainable Anomaly Detection (XOD). Anomaly detection explanations refer to the definition of a post-hoc model that explains why the initial model categorizes the objects as normal or anomalies and determines the internal causes of their predictions. In particular, explanations in unsupervised anomaly detection are equivalent to a supervised classifier ignoring the training process, finding specific patterns and subspaces in the data, and performing statistical computations in order to determine the rank of data between inliers and outliers [53]. Post-hoc explanations are useful for highly unintelligible models such as neural networks and ensemble techniques (such as decision trees, random forests, and isolation forests).

The most commonly used methods developed to explain predictions (or anomalies) from supervised or unsupervised classifiers are LIME [54] and SHAP—SHapley Additive exPlanation [55]. LIME is an explainable model that is trained on a sample data, and the model’s predictions use sparse linear models as explanations. LIME performs explanations by creating an interpretable model with local data around the neighborhood of an instance. After the model’s predictions on each sample, these sample data are weighted to the adjacency to the given instance. For example, ref. [56] proposes an explainable anomaly detection framework using SHAP for predictive maintenance in a manufacturing system, and ref. [57] explains anomalies of a deep auto-encoder model with SHAP explanations. The SHAP framework is used by [58] to explain anomalies from maritime engines with the Isolation Forest algorithm. In this study, we implement the SHAP approach to explain the traffic anomalies from the OGD sensor dataset.

SHAP assigns an importance value, called the Shapley value, inspired by game theory, to explain a particular prediction in compliance with the following properties: (i) local accuracy—the explanation model matches the original model, (ii) missingness—missing features on the original model have no impact on the explanation and (iii) consistency—transformations of the model that increase or stabilize the contribution of a feature regardless of other inputs do not lead to the decrease in this input’s attribution [55]. The prediction f(x) can be explained as:

(4) $$f\left(x\right)=g\left(x^{\prime }\right)=\phi_{0}+\phi_{i}x_{i}^{\prime }$$

where $g(x^{\prime })$ is the explanation model, *x* is the input value, $\phi_{0}$ is the baseline when all the input features are missing. According to [55], SHAP explains the output of an instance by calculating the Shapley value, i.e., the contribution of each feature to the output (prediction, anomaly):

(5) $$\phi_{i}=\sum_{S\subseteq F/i}\frac{|S|!(|F|-|S|-1)!}{|F|!}\left[f_{S\cup i}\left(x_{S\cup i}\right)-f_{S}\left(x_{S}\right)\right]$$

$\phi_{i}$ denotes the importance of the *i*th variable, *F* is the set of all variables, *S* represents all possible subsets and $f_{S}\left(x_{S}\right)$, $f_{S\cup i}\left(x_{s\cup i}\right)$ denote the output of the model before and after, including the *i*th variable to *S*, respectively.

## 3. Research Approach

The research approach of this work uses five steps, namely (1) data collection, (2) data exploration, (3) missing values analysis, (4) anomaly detection and classification, and (5) anomaly detection using multiple variables.

(1) Data collection. In this step, available traffic data from data.gov.gr (accessed on 25 October 2022) are collected using the data.gov.gr (accessed on 25 October 2022) API. These data have been produced by sensors that are positioned in the Attica Region in Greece. In addition, the position of the sensors is specified and mapped to latitude and longitude geographic coordinators in order to be able to present data in map visualizations.

(2) Data exploration. This step uses statistical analysis and visualizations to explore the collected traffic data for better understanding. Specifically, it explores the number of sensors that produced traffic data during each month and the InterQuartile Range (IQR) of the vehicles measured by each sensor. It also explores the correlation of the sensors. To this end, it employees the Pearson correlation coefficient for the number of vehicles counted each hour of the day by each sensor. The Pearson correlation is a commonly used measurement for searching correlations between time series, dividing the correlation among two variables for the product of the square of their variance. Sensors that are located nearby one another or three other sensors, i.e., their distance is lower than 2000 m, and that have a Pearson correlation coefficient greater than 0.8 are considered strongly correlated sensors. Weakly correlated sensors have a Pearson correlation coefficient greater than 0.7.

(3) Missing values analysis. Since the traffic data are dynamic data collected by sensors, there is a chance that some observations may be missing due to various reasons such as failures of sensors, network faults, and other issues. In this context, this step searches for observations that are missing from the traffic data based on two dimensions; (i) the time, and (ii) the sensors. The first case searches for the missing observations per day. To this end, we calculate for each day the number of observations that should be available for all sensors, and then we subtract this number from the number of available observations. This analysis was used to select the “best” time window for the traffic data, i.e., the time window with the least missing observations. For the second case, the total number of missing values per sensor is calculated. Additional statistical analyses are employed in order to explore the distribution of the sensors’ missing observations.

(4) Anomaly detection and classification. This step identifies traffic data anomalies, i.e., observations that do not follow the well-defined normal behavior. It also classifies detected anomalies into (i) anomalies from unusual traffic conditions (e.g., traffic accidents, weather conditions), and (ii) anomalies from sensor faults. In this work, anomaly detection comprises both the identification of (a) anomalous flow-speed correlations using flow-speed correlation analysis, and (b) deviations from the normal traffic pattern using two methods, namely, Seasonal-Trend decomposition using Loess (STL), and unsupervised learning isolation Forest (iForest), which are described in Section 2. All methods are applied to traffic data regarding the time period described by the “best” time window found in the previous step. The flow-speed correlation analysis is used to calculate the number of anomalies per sensor. Descriptive statistics are then used to describe and summarize calculated anomalies. The flow-speed correlation analysis helps to identify the “best” sensors, i.e., sensors with the lowest number of anomalies. In order to further explore the quality of data, we use the STL and iForest methods to detect anomalies in data related to the “best” sensors. For these two methods, missing values in data are imputed using the linear interpolation method. Specifically, we apply the STL decomposition in the traffic data and examine the residual curve to detect anomalies. We then specify the upper and lower fence for outlier detection by applying the IQR method in the residual curve. We experiment with multiple values in order to find the best scalar that the IQR will be multiplied by. We then classify into sensor faults and unusual traffic conditions the STL anomalies of the “best” sensors with distance between them lower than 2000 m and whose produced data are correlated based on the correlation analysis of the data exploration step. Anomalies that are simultaneously detected from correlated traffic sensors are considered unusual traffic conditions. This is based under the assumption that for proximal sensors, the unusual traffic condition will occur on the majority of sensors in the area. On the contrary, sensor faults are anomalies caused by sensor malfunctions, and hence, the adjacent traffic sensors will not capture them. Similar to the STL method, iForest is also applied to the “best” sensors’ data to detect anomalies based on their counted vehicles. The fine-tuning of the created model includes tuning the contamination hyper-parameter that represents the proportion of outliers in data. The detected anomalies are classified to sensor faults and unusual traffic conditions similarly to the STL anomalies. We compare the classifications of the two methods and find the number of measurements that were detected as anomalies by both STL and iForest methods.

(5) Anomaly detection using multiple variables. This step further explores the quality of traffic data using six additional features to create the iForest model; the average speed of the vehicles and also five temporal features, namely, (i) the weekday, a binary variable showing whether the observation is recorded on a weekday (value 1) or weekend (value 0), (ii) daylight, a binary variable to show whether the measurement was made in day (value 1) or night (value 0), (iii) the day of the week of the measurement, a number in the range 0–6 (0 = Monday and 6 = Sunday), and (iv) the hour of the day of the measurement, a number in the range 0–23. It also employs the SHapley Additive exPlanation (SHAP) framework and, specifically, the TreeSHAP variant to explain the results of the created iForest model. Two types of visualizations are used: (i) SHAP summary plots that are beeswarm plots where each dot’s position is determined by the feature on the y-axis and the Shapley value on the x-axis. The color represents the value of the feature from low to high, and (ii) SHAP dependence plots that show how a feature’s value (x-axis) impacts the prediction (y-axis) of every sample (each dot) in a dataset and how the change in SHAP values across a feature’s value range.

## 4. Case Vignettes

### 4.1. The Official Greek Open Government Data Portal

Data.gov.gr (accessed on 25 October 2022) is the official Greek data portal for Open Government Data (OGD). The latest version of the data portal was released in 2020 and provides access to 49 datasets published by the central government, local authorities, or other Greek public bodies classified in ten thematic areas, including environment, economy, and transportation.

The major update and innovation of the latest version of the Greek OGD portal was the introduction of an Application Programming Interface (API) that enables accessing and retrieving the data through either a graphical interface or code. The API is freely provided and can be employed to develop various products and services, including data intelligence applications. In order to use the API, users need to obtain a token by completing a registration process and providing personal information (i.e., name, email, and organization) as well as the reason for using the API.

The introduction of the API enables the timely provision of dynamic data that are frequently updated. The API can be used, for example, to retrieve datasets describing data related to a variety of transportation systems (e.g., road traffic for the Attica region, ticket validation of Attica’s Urban Rail Transport, and route information and passenger counts of Greek shipping companies). The frequency of the data update varies.

### 4.2. Traffic Data in the Region of Attica

Traffic data for the Attica region in Greece are collected from traffic sensor nodes, which periodically transmit information regarding the number of vehicles on specific roads of Attica along with their speed. The data are aggregated hourly in order to avoid raising privacy issues. Data are updated hourly with only one hour delay.

We used the API provided by data.gov.gr (accessed on 25 October 2022) and collected 4,230,819 records for a 22-month period, i.e., from 5 November 2020 to 31 June 2022. Each record includes (a) the unique identifier of the sensor (e.g., MS834), (b) the road in which the sensor is located along with (c) a detailed text description of its position, (d) the date and time of the measurement, (e) the absolute number of the vehicles detected by the sensor during the hour of measurement, and (f) their average speed in km per hour. The exact position of the sensor is a text description in the Greek language and usually provides details, including whether the sensor is located on a main or side road, or on an exit or entrance ramp, the direction of the road (e.g., direction to center), and the distance to main roads (e.g., “200 m from Kifisias avenue”).

The collected traffic data have been produced by 425 sensors. We manually mapped the position of the sensors to latitude and longitude geographic coordinators in order to be able to present data in a map visualization. Specific position details are missing for one sensor (i.e., the sensor with identifier “MS339”), making it impossible to find its exact coordinates.

**Figure 1:**
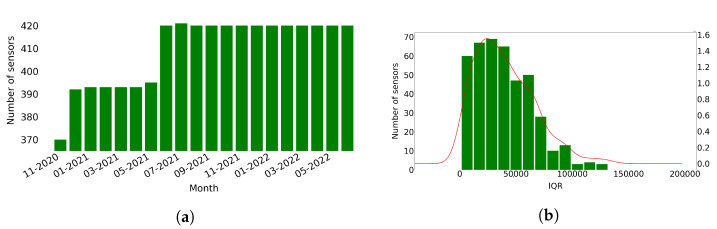
Number of sensors that produce traffic data per day and distribution of the IQR of the number of vehicles measured by each sensor. (**a**) Number of sensors that produce traffic data each month. (**b**) Distribution of the IQR of the vehicles measured by each sensor.

According to the data, the sensors did not start producing traffic data at the same time, and few of them stopped before the end of the period. Figure 1a presents the number of sensors that produced traffic data each month. Most sensors (370 or 87%) produce data from the first month of the data (November 2020), and then the number of sensors producing data gradually increases to reach the 420 sensors in June 2021. Specifically, during June 2021, 25 new sensors were introduced and, in the same month, two sensors stopped producing data. A new sensor also started producing data in July 2021, resulting in 421 sensors, and then a sensor stopped producing data on August 2021, resulting again in 420 sensors. Finally, in December 2021, two new sensors started producing data, and two stopped keeping the number of sensors stable to 420 until the end of June 2022. We excluded from this work the data related to sensors that stopped producing data (namely sensors with IDs ‘MS136’, ‘MS137’, ‘MS858’, ‘MS1000’, and ‘MS1001’). Finally, we gathered a total of 4,228,021 observations.

We then calculated the InterQuartile Range (IQR) of the counted vehicles measured by each sensor. Figure 1b shows the right-skewed distribution of IQR, meaning that few of the sensors probably counted large number of vehicles.

We also explored the correlation between all sensors by calculating the Pearson correlation coefficient for the number of vehicles counted each hour of the day by each sensor. In order for two sensors to be correlated, their distance should be lower than or equal to 2000 m. Based on this criterion, out of the 425 sensors, 417 have at least three correlated sensors (Table 1), while the average number of correlated sensors is 40.76. From these sensors, 404 have a Pearson correlation coefficient greater than 0.8, while 410 have a Pearson correlation coefficient greater than 0.7. Finally, there are six sensors that have one or two correlated sensors, while two sensors are isolated.

## 5. Missing Values

In this section, we search for observations that are missing from the traffic data based on two dimensions; (i) the time and (ii) the sensors. In the first case, we calculate the missing values per day, whilst in the second, the missing values per sensor.

**Figure 2:**
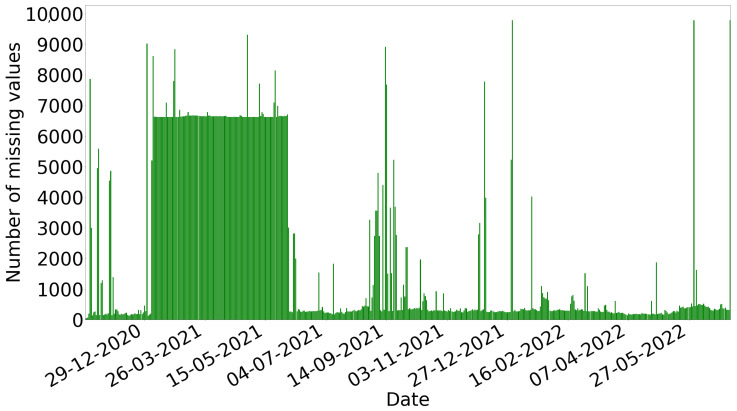
Number of observations that are missing per day.

Considering all the sensor measurements in the time frame between 5 November 2020 and 30 June 2022 and the time interval each of the 420 sensors was producing data, the number of the total potential observations would be 5,295,504. However, 1,067,483 observations (or 20.16%) are missing. Figure 2 presents the number of missing observations per day. The number of missing observations are increased until the end of May 2021. However, from June 2021, there is a significant decrease in the number of observations that are missing. Finally, the number of missing observations starts to eliminate after January 2022 and, specifically, after 2 January 2022. We select the time period from 2 January 2022 to 23 June 2022 as the “best” time period. This time period includes 75,425 missing observations.

**Figure 3:**
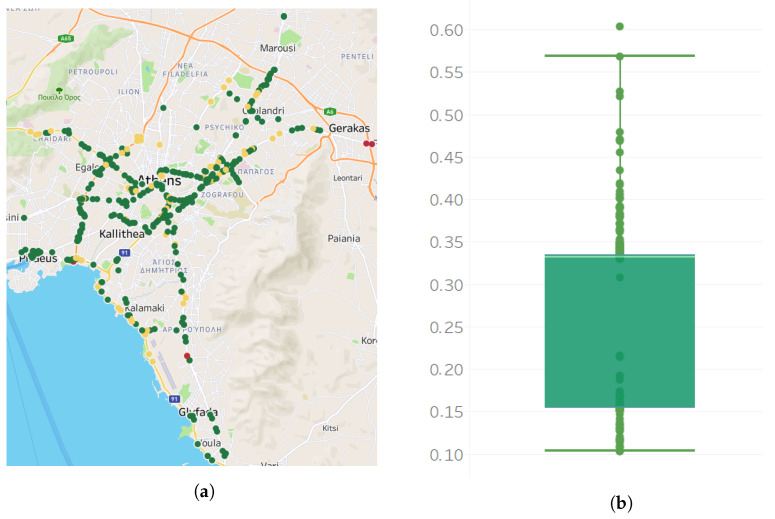
Map of sensors and boxplot for the missing values per sensor. (**a**) View of the 420 sensors on a map. Each point on the map represents a sensor. Red color marks represent sensors with higher percentages of missing values, green marks sensors with low percentages of missing values. (**b**) Percent of missing values per sensor in a boxplot showing the lower (Q1) and upper (Q3) quartile, the median and mean values. Data falling outside the lower (Q1)–upper (Q3) quartile range are plotted as outliers of the data.

We also calculate the percent of missing observations for each sensor from 5 November 2020 to 30 June 2022. Figure 3 presents the street map with the 420 sensors and also the boxplot, which presents the distribution of the missing observations per sensor. The sensors are positioned on 93 main roads in the region of Attica. In Figure 3a, a mark is displayed over each sensor’s longitude and latitude in the region of Attica. The colors of the marks indicate the percent of missing observations of the sensor; red color marks represent sensors with higher percentages of missing observations, green marks sensors with lower percentages of missing observations, and yellow marks sensors with intermediate percentages of missing observations. In addition, the boxplot in Figure 3b presents the distribution of the number of missing observations per sensor. The median percent of missing observations is 33.1%, meaning that half of the sensors have less than or equal percentages of missing observations to the median, and half of the sensors have greater than or equal percentages of missing observations to it. In total, 50% of the sensors have a percentage of missing observations in the range of 15.5–33.43% (interquartile range box). In addition, according to the whiskers of the boxplot (bottom 25% and top 25% of the data values, excluding outliers), the percent of missing observations of each sensor may be as low as 10.3% and as high as 56.8%. In addition, based on our calculations, only eight sensors have less than 10% missing observations. Finally, only one sensor has a percentage of missing observations above 60%.

## 6. Anomaly Detection

In this section, we detect the anomalies in the traffic data based on three anomaly detection methods, namely (i) flow-speed correlation, (ii) seasonal-trend decomposition with Loess, and (iii) isolation forest. The time window we select to perform the above analyses is the “best” time window starting from 2 January 2022 to 23 June 2022. In this time window, the dataset includes 1,679,898 records with measurements produced by 420 sensors.

### 6.1. Flow-Speed Correlation Analysis

We first calculate the number and percentages of anomalies per sensor based on the flow-speed correlation analysis method described in the research approach section (Section 3). In order to be able to calculate the number of vehicles that can pass in all lanes, we manually found the number of lanes that each sensor tracks and mapped them to the records. We discovered 1,230,928 observations that count more vehicles than the number calculated by the filter (59.4% of total potential observations). We also calculate the number of anomalies per sensor. This number ranges from 0 to 3853 anomalies. In addition, the mean number of detected anomalies per sensor is 2937.8, while the median is 3264 anomalies per sensor. Table 2 presents the descriptive statistics for the anomalies detected per sensor.

The flow-speed correlation analysis showed that the vast majority of sensor recordings of the OGD dataset exceed the threshold value. For example, sensors MS792 and MS108 have 92.04% and 91.58% of anomalies, respectively. Only nine sensors have less than 10% anomalies based on the flow-speed correlation analysis (Table 3). We, henceforth, consider them as the “best” sensors for the selected time window. For these sensors, we calculate the number of anomalies based on the STL decomposition and iForest methods to further explore the quality of the traffic OGD. For both of these methods, identified missing values were imputed using the linear interpolation method.

### 6.2. Anomaly Detection and Classification with STL Decomposition

**Figure 4:**
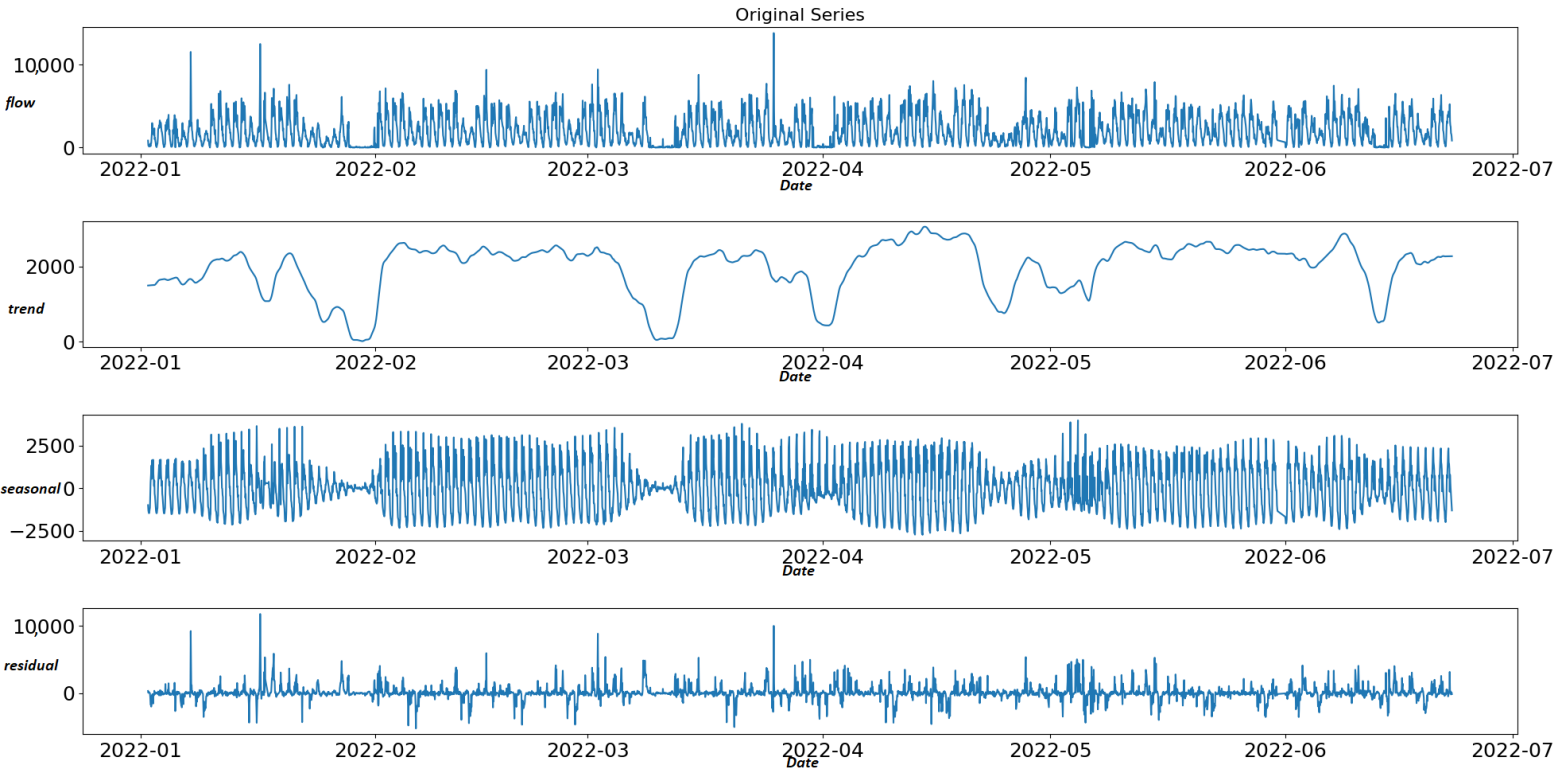
STL decomposition of time series of sensor MS734.

The STL decomposition is applied to aggregated traffic data for the corresponding time window between 2 January 2022 and 23 June 2022 (4152 h). STL detects 4720 anomalies related to the nine “best” sensors (12.6% of 37,368 total records) of Table 3. Figure 4 shows an example of STL decomposition related to the observations of one sensor (sensor “MS734”) for the selected time window. For anomaly detection, we examine the last curve of the STL method, the residual curve. Therefore, we apply the IQR method in the residual curve to specify the upper and lower fence for outlier detection. Observations above the upper limit and below the lower limit are considered anomalies. Finally, we set the scalar multiplied with IQR to three after a set of experiments. The upper and lower limits of the residual curve are defined using the following formula:

(6) $$Upperlimit=Q_{3}+3IQR$$

(7) $$Lowerlimit=Q_{1}-3IQR$$

**Figure 5:**
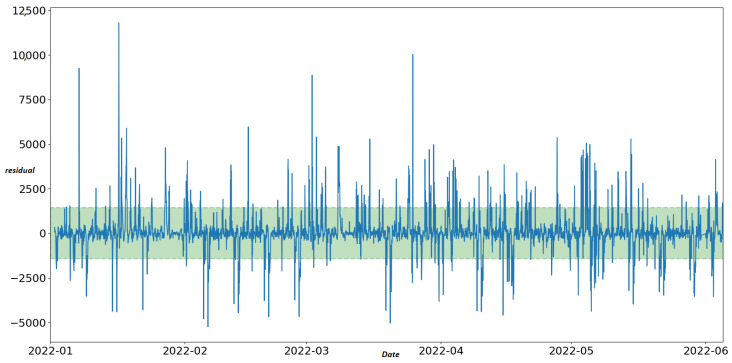
Residual curve of sensor “MS734” with the upper and lower fences of the IQR method. The green dots represent unusual traffic conditions. Red dots represent sensor faults.

The residual curve for sensor “MS734” depicted in Figure 5 shows that anomalies of this particular sensor are detected both in positive and negative peaks of the remainder component of STL.

Finally, we classify the STL traffic anomalies into sensor faults and unusual traffic conditions. We only classify anomalies detected by sensors with a distance between them lower than 2000 m and whose produced data are correlated based on the correlation analysis made in Section 4.2. Among the “best” sensors, only two pairs, i.e., “MS941” and “MS121”, and “MS145” and “MS734”, satisfy the previous criteria. Specifically, the distance between “MS941” and “MS121” is 1538.34 m, while the Pearson correlation between their produced data is 0.835128. In addition, the distance between “MS145” and “MS734” is 1333.49 m, while the Pearson correlation between their produced data is 0.783.

Table 4 shows the total number of anomalies detected by the STL decomposition method for the nine reliable sensors and the number of STL anomalies classified as unusual traffic conditions and sensor faults. The classification shows that unusual traffic conditions and sensor faults are almost equally distributed within the total number of anomalies. We further explored the classified anomalies. In total, 97% of unusual traffic conditions were detected during the day (between 07:00 and 20:00) and 56% were classified on weekdays. In total, 90% of sensor faults were detected in daylight, while 62% were detected on weekdays.

**Figure 6:**
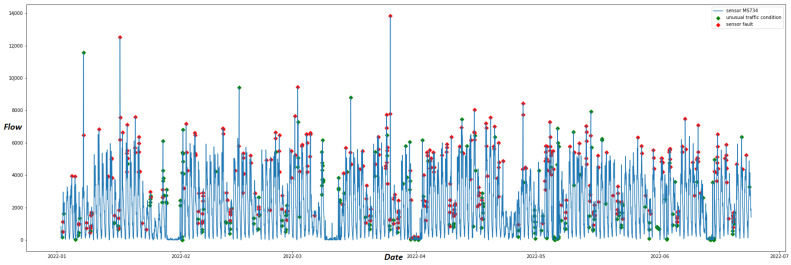
Time series of sensor “MS734” with anomalies detected by STL decomposition. Red dots are anomalies classified as “sensor faults”, and green dots are anomalies classified as “unusual traffic conditions”.

Figure 6 depicts the anomalies detected in the data produced by sensor “MS734” on the residual curve upon the original time series. Among these anomalies, unusual traffic conditions are depicted as green dots. These anomalies are also detected by STL on the correlated sensor “MS145”. On the contrary, anomalies that are not recorded by proximal sensors on the same timestamp are considered sensor faults and depicted with red dots.

### 6.3. Anomaly Detection and Classification with Isolation Forest

Next, we apply the isolation Forest (iForest) algorithm to the OGD traffic dataset. Similar to the STL method, iForest is implemented on the same time window between 2 January 2022 and 23 June 2022. The algorithm is deployed using python’s Scikit-learn machine learning library (https://scikit-learn.org/stable/modules/generated/sklearn.ensemble.IsolationForest.html/ accessed on 25 October 2022). Furthermore, the only hyper-parameter of the model needed to be tuned is the contamination parameter, which represents the proportion of outliers in the dataset. Similar to other ensemble learning techniques, iForest is made up of a large number of decision trees. The number of isolation trees is represented by the parameter $n_{estimators}$, which is set to 100 for all the time series in this experiment. Finally, the parameter $max_{samples}$, which is the number of random samples from the original dataset that will be created for the isolation trees, is set to 256. The iForest algorithm detects 4353 anomalies out of 37,368 records (11.6%) of the data from the nine sensors.

Similar to the STL method, we apply the classification method to the data of the correlated sensors “MS121”, “MS145”, and “MS734” and “MS941”. Table 5 shows the total number of anomalies (out of 4152 records per sensor) that were identified by iForest and the classified anomalies among the correlated sensors. The majority of anomalies are classified as sensor faults. Especially for sensors “MS121” and “MS941”, 78.7% and 89.7% of the detected anomalies, respectively, are classified as sensor faults. We further explored the classified anomalies. A vast majority of classified unusual traffic conditions, almost 98%, were detected during daylight (between 07:00 and 20:00) and 55% of them during weekdays. Finally, 81% of the detected sensor faults were recorded both in daylight hours and on weekdays.

**Figure 7:**
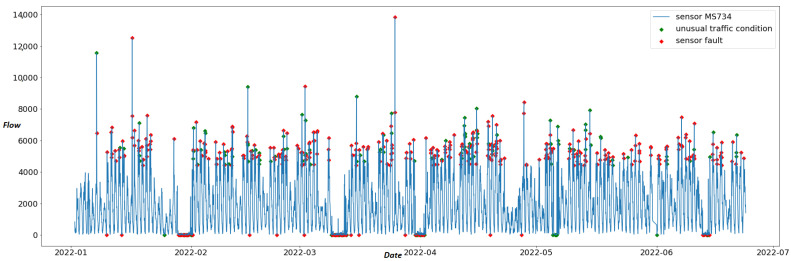
Time series of sensor “MS734” with anomalies detected by the iForest algorithm. Red dots are anomalies classified as “sensor faults” and green dots are anomalies classified as “unusual traffic conditions”.

Figure 7 displays the anomalies detected by iForest classified as unusual traffic conditions (green dots) and as sensor faults (red dots) for sensor “MS734”.

In Table 6, the anomalies detected by STL and iForest are compared. For the nine “best” sensors, each column points out the (i) total overlaps, (ii) overlaps of unusual traffic conditions, and (iii) overlaps in sensor faults of the anomalies detected by the two methods. We observe that only sensor “MS944” has a high value of overlap, with 420 anomalies detected by both anomaly detection algorithms. On the contrary, our methods detect only 65 and 88 common anomalies for sensors “MS121” and “MS941”, respectively. According to this table, the overlaps regarding the classification of anomalies are very low between the two methods. Only sensor MS734 has 111 overlaps on sensor faults. The major differences in the two proposed methods are also depicted in Figure 6 and Figure 7. The iForest technique detects anomalies mainly on the low and high peaks of the time series since the algorithm manages to classify anomalies due to their significant deviation from the normal pattern. On the contrary, as Figure 6 illustrates, STL applies anomaly detection on the residual curve with the IQR method thinking of the time series as a combination of trend, seasonality and remainder. Thus, it highlights anomalies taking into consideration trend and seasonality effects of the time series. These might be significant reasons for the small number of overlaps between the two methods, as Table 6 suggests. However, our experiments are conducted only on 9 out of 420 sensors of the traffic network due to the flow-speed correlation analysis results. Thus, we need a more reliable dataset with a greater number of sensors to correctly compare the two methods.

## 7. Anomaly Detection Using Multiple Variables

In order to further explore the quality of the OGD traffic dataset, we train the isolation Forest (iForest) algorithm on additional features and make explanations using the SHapley Additive exPlanation (SHAP) framework to interpret the classification of anomalies. Our goal is to explore specific patterns among the traffic dataset that may lead to anomalies and, hence, deteriorate its quality. In this direction, the following explanations do not interpret the exact reasons in anomalies but rather give an estimation of what the indicators are that might contribute to a traffic anomaly. The iForest algorithm is suitable for high-dimensional anomaly detection involving multiple features during the partitioning process. The main difference from the one-feature implementation is that the algorithm randomly selects a feature every time it partitions a data point. Then the model selects a split value between the minimum and maximum values of the selected feature. For that reason, we calculated four temporal features from our time series: (i) the weekday, a binary variable showing whether the observation is recorded on a weekday (value 1) or weekend (value 0), (ii) daylight, a binary variable to show whether the measurement was made in the day (value 1) or night (value 0), (iii) the day of the week of the measurement, a number in the range 0–6 (0 = Monday and 6 = Sunday), and (iv) the hour of the day of the measurement, a number in the range 0–23. Finally, we also add the average speed of vehicles measured by the traffic sensors for one hour.

The iForest algorithm is explained using the SHAP values of every data instance obtained by the nine “best” sensors. For the calculation of SHAP values, the TreeSHAP model [55] was used to provide an anomaly explanation.

**Figure 8:**
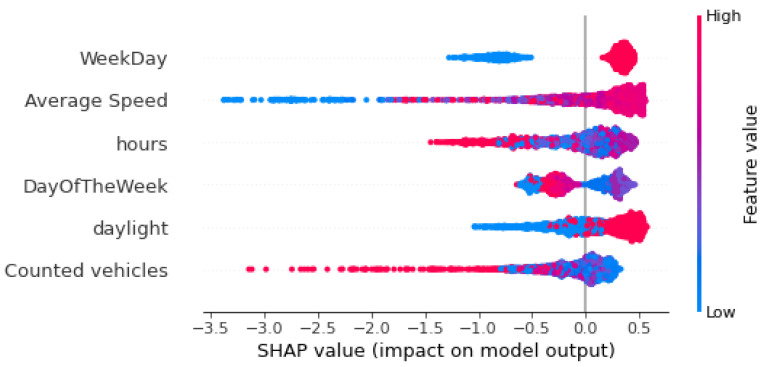
Distribution of SHAP values for each feature, sensor “MS734”.

**Figure 9:**
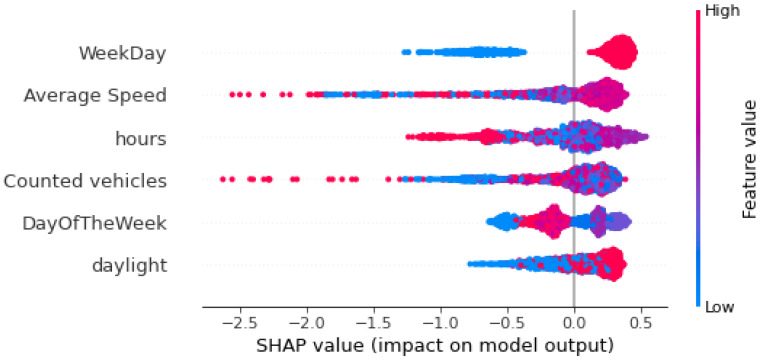
Distribution of SHAP values for each feature, sensor “MS941”.

In order to understand how several variables affect the detection of anomalies, we created a SHAP summary plot. Figure 8 and Figure 9 show the global interpretation of Shapley values for sensors MS734 and MS941, respectively. The plots present the features with the highest impact on the output of the model in descending global importance. The X-axis shows the Shapley values of each measurement for every feature; Shapley values below zero mean that the model classifies a data instance as an anomaly, while Shapley values greater than zero mean that iForest classifies a data instance as normal. In these figures, each dot represents the Shapley value of every data instance (every observation). Each dot is also colored by the value of that feature from high to low, with red dots representing high values and blue dots as low values of a feature. According to the summary plots in Figure 8 and Figure 9, “WeekDay” and “Average Speed” are the two features with the highest impact for anomaly detection. “WeekDay” is a feature that indicates whether the measurement took place on a workday “WeekDay” = 1) or during the weekend (“WeekDay” = 0). Therefore, high values of this feature indicate that the recording happened on a weekday. Data points that are recorded on weekends are represented in the summary plots with blue color and have low (negative) Shapley values, meaning that anomalies are more likely detected on weekends. On the contrary, data instances that are recorded in a weekday are presented in the summary plots with red color and have high (positive) Shapley values, indicating that normal points are more likely detected on a weekday. We are also interested in long left tails that form the beeswarm plots because they correspond to extreme anomalies (extremely negative Shapley values). In the case of sensor MS734 (Figure 8), extremely low values of “Average Speed” correspond to very low Shapley values meaning that low average speeds are indicators of extreme anomalies. Moreover, in the same plot, high values of “Counted vehicles”, a feature with low global importance, imply the existence of very high anomalies for both sensors. In the same way, both summary plots illustrate that blue dots of variable “daylight” are mostly gathered on the negative part of X-axis, implying that the algorithm detects anomalies during night hours. Finally, “Day of the Week” does not display a clear pattern regarding its influence on the model’s outcome.

**Figure 10:**
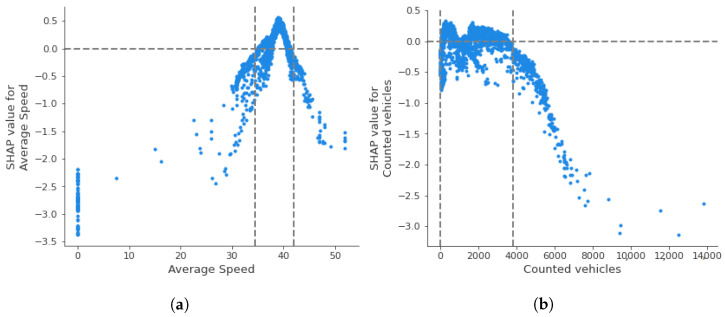
SHAP dependence plots of sensor “MS734”. (**a**) Average speed vs. SHAP values. (**b**) Traffic flow vs. SHAP values.

In order to fully understand the relationship between a feature’s values and the model’s outcomes and estimate the influence of variables on anomaly detection, we also create SHAP dependence plots. A dependence plot depicts every data instance (i.e., every row) as a blue dot. These dots form a scatter plot of the feature’s raw values versus the corresponding SHAP values. Figure 10 illustrates dependence plots for the two measured variables of the traffic sensors: average speed and counted vehicles for sensor MS734. SHAP values above the y = 0 line lead to the detection of normal points, whereas those below it are considered anomalies. For instance, the pattern of the Figure 10a plot indicates that sensor measurements with zero average speed are considered anomalies. Furthermore, there is a range of average speed between 33 and 42 km/h, where the corresponding SHAP values are positive, indicating that sensor measurements between this range are more likely considered normal. Figure 10b shows the number of counted vehicles measured by sensor MS734 and their corresponding SHAP values. For this sensor, measurements with more than 3800 vehicles per hour have a significant decrease in SHAP values, clearly indicating anomalous traffic behavior. Moreover, recordings with counted vehicles between zero and 3800 vehicles per hour can be both normal and anomalous.

## 8. Discussion

During the last two decades, the Open Government Data (OGD) phenomenon has rapidly evolved. In these years, numerous OGD portals have already opened up their data for free reuse, and the economic and social potential of OGD has significantly increased. In order to facilitate the involvement of OGD in the creation of data intelligence applications, various technologies have been used. Linked data is a technological paradigm that was proposed early on [1] and has already been adopted by many OGD portals developed by, e.g., the Scottish Government (https://statistics.gov.scot/ accessed on 25 October 2022) and the UK Department for Communities and Local Government (DCLG) (http://opendatacommunities.org/ accessed on 25 October 2022). Linked data not only facilitated the integration of data both inside and across data portals but also ensured the provision of high-quality data. This is of vital importance in specific types of OGD, such as statistical data, where data are described in different granularity levels [59]. The provision of high-quality statistical data that can be easily integrated enables the emergence of added-value scenarios that can be easily implemented. Indeed, in our previous work, we employed linked statistical data from the Scottish OGD portal to facilitate policy-making related to house prices in Scotland using machine learning and explainable artificial intelligence technologies [20].

Today, however, the amount of Open Government Data (OGD) that are being produced and disseminated is exponentially growing while new types of data are also being generated, such as dynamic OGD (e.g., traffic data generated by sensors), which were recognized as an important part of OGD. At the same time, the recent emergence of innovative data analysis and exploitation methods (e.g., artificial intelligence including machine learning) opens up new opportunities for exploiting OGD. It is, hence, important to define and address the new challenges that are related to the exploitation of these new types of data using innovative methods in order to extract the potential that previous types of data and methods cannot.

In this context, this paper focused on traffic data, which is a prominent example of high-value data, and explored the quality of Attica traffic data. To the authors’ knowledge, this is the first time a study has explored the quality of OGD by studying their data and not the metadata provided by OGD portals. For example, previous works are based on assessing metadata, such as the data formats available (including whether they are provided in a machine readable format) [4,6], the accuracy of the metadata (i.e., whether the provided metadata are correct) [4,60], the timeliness of the metadata (i.e., whether metadata are up-to-date) [61], and the discoverability, reusability and accessibility of the datasets [4,62].

The data used in this work are provided by data.gov.gr (accessed on 25 October 2022)), the official Greek OGD portal, through an API, ensuring their immediate availability and are updated hourly with only a one hour delay. The research approach includes steps that can applied to assess the quality of more traffic datasets provided by other OGD portals. We found that considering the time frame between 5 November 2020 and 30 June 2022, and the number of days each one of the 420 sensors was producing data, 20.16% of the observations are missing. In addition, 50% of the sensors have a percentage of missing values in the range 15.5–33.43% (interquartile range box). We also used flow-speed correlation analysis to detect anomalous flow-speed correlations and two methods, namely Seasonal-Trend decomposition using Loess (STL) and unsupervised learning isolation Forest (iForest), to detect deviations from the normal traffic pattern. The flow-speed correlation analysis found that the mean percent of anomalies per sensor is 71.1%. Only nine sensors have less than 10% anomalies based on the flow-speed correlation analysis. For these nine sensors, STL decomposition detected 4720 anomalies (12.6%) and isolation Forest, 4353 anomalies (11.6%). However, the comparison of the two methods showed that there a few overlaps between the proposed methods. STL manages to detect anomalies by taking into consideration seasonality and trend effects, while iForest mainly detects the high and low peaks of the corresponding time series. As a result, using both methods to detect anomalies appears to be a good strategy. Furthermore, our proposed classification method showed that both unusual traffic conditions and sensor faults are detected mainly on weekdays and during daylight hours, while the iForest algorithm manages to detect more sensor faults than the STL method. Finally, in order to have an estimation of the factors that influence traffic anomalies, we implemented the SHAP framework upon the iForest model. The results showed that average speed and the day during the recording (weekday or weekend) are the most significant factors. Moreover, we found that very low values of average speed have a high contribution to the identification of anomalies, while sensor measurements with counted vehicles per hour greater than 3800 lead to the classification of extreme anomalies.

## 9. Conclusions

The findings of this study demonstrate that, while traffic data from the Greek OGD portal may be retrieved promptly via the API and are constantly updated, they confront major quality difficulties. However, this may not be the case for all OGD portals’ dynamic data. We believe that further research in the dynamic data of other OGD sites will disclose datasets of higher quality that might be potentially used in added-value scenarios. In any case, the exploration of big and real-time data provided by OGD portals will enable identifying and addressing organizational and technical challenges that hamper the effective dissemination of high-value government data.

## Figures and Tables

**Table 1:** Correlation of sensors based on the counted number of vehicles counted each hour of the day. Correlated sensors have distance ≤ 2000 m and more than 3 correlations. Strongly correlated sensors have distance ≤ 2000 m and more than 3 correlations with Pearson coefficient ≥ 0.8. Weakly correlated sensors have distance ≤ 2000 m and more than 3 correlations with Pearson coefficient ≥ 0.7. Loosely correlated sensors have distance ≤ 2000 m and 1 or 2 correlations.

	Correlation
Total number of sensors	425
Correlated sensors	417
Strongly correlated sensors	404
Weakly correlated sensors	410
Loosely Correlated sensors	6
Isolated sensors	2

**Table 2:** Descriptive statistics considering the number of anomalies per sensor using the flow-speed correlation analysis.

	Number of Anomalies	Mean Number of Anomalies
mean	2937.8	71.1
standard deviation	821.7	19.9
min	0	0
first quartile	2824	68.4
median	3264	79
third quartile	3416	82.75
max	3853	93.3

**Table 3:** Traffic sensors with less than 10% detected anomalies using the flow-speed correlation analysis.

Sensor ID	Hours of Anomaly	Percentage of Anomalies (%)
MS346	378	9.1
MS121	321	7.73
MS941	308	7.41
MS309	295	7.10
MS944	178	4.28
MS145	48	1.15
MS134	18	0.43
MS502	14	0.33
MS734	8	0.19

**Table 4:** Anomalies detected by the STL decomposition.

Sensor ID	STL Anomalies	Unusual Traffic Conditions	Sensor Faults
MS121	359	179	180
MS134	502	n/a	n/a
MS145	464	256	208
MS309	679	n/a	n/a
MS346	489	n/a	n/a
MS502	384	n/a	n/a
MS734	615	256	359
MS941	454	179	275
MS944	774	n/a	n/a

**Table 5:** Anomalies detected by the iForest algorithm.

Sensor ID	iForest Anomalies	Unusual Traffic Conditions	Sensor Faults
MS121	287	61	226
MS134	441	n/a	n/a
MS145	494	214	280
MS309	619	n/a	n/a
MS346	535	n/a	n/a
MS502	316	n/a	n/a
MS734	575	214	361
MS941	385	61	324
MS944	701	n/a	n/a

**Table 6:** Comparison of STL decomposition and isolation forest anomaly detection algorithms.

Sensor ID	Total Overlap	Unusual Traffic Conditions	Sensor Faults
MS121	88	15	52
MS134	222	n/a	n/a
MS145	115	34	45
MS309	180	n/a	n/a
MS346	177	n/a	n/a
MS502	176	n/a	n/a
MS734	211	34	111
MS941	65	15	29
MS944	420	n/a	n/a

## Data Availability

Publicly available datasets were analyzed in this study. These data can be found here: (https://data.gov.gr/datasets/road_traffic_attica/) (accessed on 25 October 2022).

## Abbreviations

The following abbreviations are used in this manuscript:

API	Application Programming Interface
HVD	High-Value Datasets
OGD	Open Government Data
STL	Seasonal-Trend decomposition using Loess
